# Translation, Cross-Cultural Adaptation, and Validation of the Pain Sensitivity Questionnaire in Dutch Healthy Volunteers

**DOI:** 10.1155/2020/1050935

**Published:** 2020-07-23

**Authors:** Regina L. M. Van Boekel, Hans Timmerman, Ewald M. Bronkhorst, Ruth Ruscheweyh, Kris C. P. Vissers, Monique A. H. Steegers

**Affiliations:** ^1^Department of Anesthesiology Pain and Palliative Medicine, Radboud University Medical Center, Nijmegen, Netherlands; ^2^Department of Anesthesiology, Pain Center, University Medical Center Groningen, Groningen, Netherlands; ^3^Department for Health Evidence, Radboud University Medical Center, Nijmegen, Netherlands; ^4^Department of Neurology, Ludwig Maximilians University of Munich, Munich, Germany; ^5^Department of Anesthesiology, Amsterdam University Medical Center, Location VU, Amsterdam, Netherlands

## Abstract

An increased sensitivity to painful stimuli has been proposed to be related to the development of chronic pain. Therefore, assessment of individual pain sensitivity is useful in clinical practice. However, experimental pain testing may be uncomfortable for patients and requires specific equipment. The Pain Sensitivity Questionnaire (PSQ) has been developed to facilitate assessment of pain sensitivity. In this study, we aimed to translate and cross-culturally adapt the PSQ from its published German and English versions into the Dutch language and to assess validity of the PSQ in healthy volunteers. After translation and cross-cultural adaptation of the PSQ following international guidelines, we validated the PSQ in 394 healthy volunteers by comparing the PSQ-values with two different experimental pain tests: electrical pain tolerance (EPT) and pressure pain threshold (PPT). In addition, ratings of pain intensity during these tests were obtained on the numerical rating scale (NRS, 0–10). We found that the reliability of the PSQ based on internal consistency was good (Cronbach's alpha 0.90). PSQ-scores, adjusted for age and sex, were statistically significant and weakly inversely correlated to EPT (PSQ-moderate: rho = −0.24, *p*=0.007; PSQ-total: rho = −0.22, *p*=0.016). No statistically significant correlation between PSQ-scores and PPT was found. Concerning the pain scores, PSQ-scores were weakly to moderately correlated to EPT-NRS (PSQ-minor: rho = 0.21, *p*=0.021; PSQ-moderate: rho = 0.22, *p*=0.016; PSQ-total: rho = 0.23, *p*=0.009) as well as PPT-NRS (PSQ-minor: rho = 0.32, *p* < 0.001; PSQ-moderate: rho = 0.36, *p* < 0.001; PSQ-total: rho = 0.37, *p* < 0.001). Therefore, we concluded that the Dutch version of the PSQ is culturally appropriate for assessing self-reported pain sensitivity in healthy volunteers.

## 1. Introduction

Increased pain perception and pain sensitivity may be related to the development of chronic pain [1]. The need for a proper assessment of individual pain sensitivity is well known, since pain sensitivity may influence initial pain experiences and treatment outcome [2, 3]. Experimental determination of a patients' pain sensitivity is possible by quantitative sensory testing (QST) using different stimulation modalities such as thermal, mechanical, ischemic, or electrical stimulation [4, 5]. Ruscheweyh and coworkers developed the Pain Sensitivity Questionnaire (PSQ) to easily measure patients' pain sensitivity in daily clinical practice without experimental determination of patient's pain [6].

The PSQ is based on pain intensity rating of imagined painful daily life situations. The validity of the PSQ to measure perceived pain sensitivity has been shown by significant correlations with experimental pain intensity, both in healthy subjects and in patients with chronic pain [6, 7]. In contrast, correlations with experimental pain thresholds were lower or absent [6]. Besides the original version in the German language [6], the PSQ is translated into and validated in English [8], French [9], Polish [10], Korean [11], Persian [12], Mandarin [13], and Norwegian [14]. Moreover, the PSQ has been used to identify pain sensitivity in several studies: to predict surgical success in patients with lumbar disc herniation [12, 15] and lumbar stenosis [16], to predict postoperative pain and development of chronic pain in patients after spine surgery [17], and to predict acute postoperative pain following surgery for breast cancer [18]. It is also used to understand ocular discomfort and dryness [19], in epidemiological health research [20], to explore associations with widespread pain in patients with shoulder pain [21], and to discriminate in the pain behavior of patients with chronic pain with or without central sensitization [22].The correlation between ethnic differences and clinical pain response was also studied by use of the PSQ [23].

According to these previous results, the PSQ might be a clinical useful instrument to screen patients' pain sensitivity in the Netherlands. However, in order to use this tool in a Dutch population, the PSQ needs to undergo a translation into the Dutch language. The next step is to validate the new Dutch version of the PSQ in a population of healthy volunteers. Therefore, the aim of this study was to translate and cross-culturally adapt the PSQ from the published English and German versions into the Dutch language and to validate this instrument in a large group of healthy volunteers by comparing PSQ-scores with the results of experimental pain testing.

## 2. Methods

### 2.1. Translation and Cross-Cultural Adaptation of the PSQ to the Dutch Language

**Figure 1:**
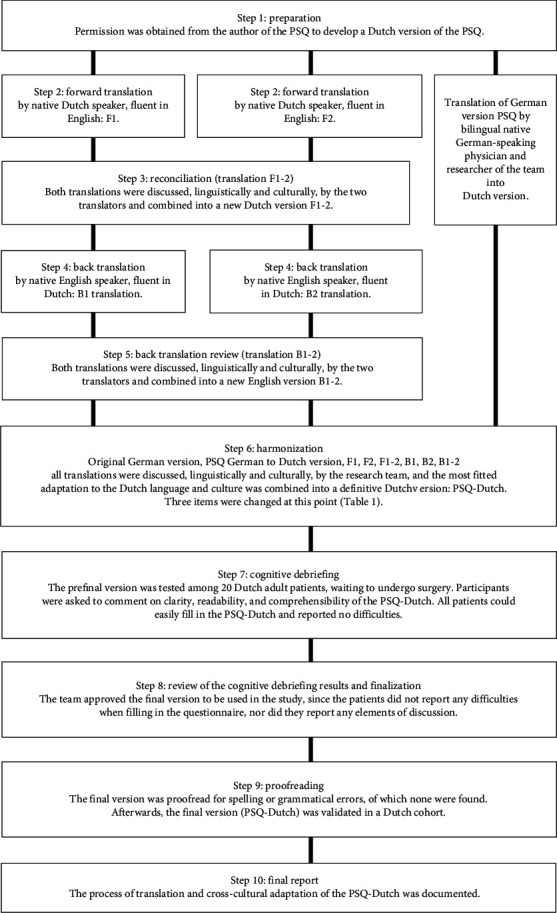
Flow diagram of the translation and cross-cultural adaptation process.

Permission was obtained from the initial developers (Ruscheweyh and coworkers) to develop and cross-culturally adapt a Dutch version of the PSQ and to validate this Dutch version of the PSQ against experimental pain testing. The validated German and English versions of the PSQ from the study of Ruscheweyh and coworkers [6, 8] were translated into the Dutch language according to international guidelines [24] (see Figure 1). A native German speaking bilingual physician and researcher translated the original German version into Dutch. On the basis of the English version, two independent official bilingual, native Dutch speaking translators, of which one had a medical background, independently translated the PSQ from English into Dutch. The two Dutch versions were put together and after careful consideration combined into one new Dutch version. Afterwards, this Dutch version was back translated into English by two independent official bilingual English translators, of which one had a medical background, and compared to the original English version. This new English version was compared to the original English version, and some items were discussed (see Table 1). To assess face validity and a qualitative evaluation, a pilot test was conducted with twenty patients waiting to undergo different kinds of surgery, such as orthopedic, abdominal, or gynecological surgery. Patients did not report any difficulties when filling in the questionnaire, nor did they report any elements of discussion. Because the cognitive debriefing left the PSQ-Dutch unchanged, the questions in the questionnaire were considered to be adequate and understandable. The native German speaking bilingual researcher compared the original German PSQ with his own translation and the final PSQ-Dutch. The PSQ-Dutch was also found to be similar to the original German questionnaire. In conclusion, the pilot test phase of the PSQ-Dutch showed no necessities for adjustments (see Supplement S1 for the Dutch version of the Pain Sensitivity Questionnaire (PSQ-Dutch)).

### 2.2. Validation of the PSQ

#### 2.2.1. Design

This observational study took place in 2016 during “Lowlands,” a 3-day festival with special attention for science, in Biddinghuizen, Netherlands. The study was conducted according to the Helsinki Declaration and was approved by the local research ethics committee on human research (Medical Review Ethics Committee Region Arnhem-Nijmegen) beforehand. It is registered as file number CMO: 2016–2784.

#### 2.2.2. Participants

Participants were recruited by members of the research team when passing by the study facility. All participants were 18 years or older and could speak and read the Dutch language. All participants gave a written informed consent. Participants received no financial compensation for their cooperation. Exclusion criteria were as follows: use of pain killers in the last 12 hours, use of antidepressants, pain of the arm, neck or shoulder (uni- or bilaterally), cardiac disease, psychiatric or neurological disease, injury to the forearms or hands, Raynaud disease, pregnancy, blood alcohol content (BAC) of >220 *μ*g/l, or use of recreational drugs in the past 24 hours. Participants were tested for their blood alcohol content by a physician, via breath analysis.

#### 2.2.3. Test Methods

*(1) Pain Sensitivity Questionnaire*. Participants filled in the questions of the PSQ directly after being included in the study. The PSQ consists of 17 questions, which describe situations in daily life. Respondents score their pain intensity of the described situation ranging from 0 (not painful) to 10 (strongest pain imaginable) on a numeric rating scale. Fourteen questions represent situations that the majority of healthy subjects consider painful. Three questions represent nonpainful situations as perceived by most people. These three questions are used as a nonpainful sensory reference for the participants and are not included in the final scoring. A diversity of pain modalities such as hot, cold, sharp, and blunt, as well as different parts of the body such as head, upper extremity, and lower extremity, are included in the questionnaire. The PSQ-total score is formed as the average of the fourteen “painful” items of the PSQ, all ranging from 0 to 10. Two subscores have been derived from factor analysis, the PSQ-minor (including items that on average are associated with minor pain) and the PSQ-moderate score (including items that on average are associated with moderate pain) [6]. The PSQ-minor consists of the mean rating of questions 3, 6, 7, 10, 11, 12, and 14. The PSQ-moderate consists of the mean rating of questions 1, 2, 4, 8, 15, 16, and 17. In the original study of Ruscheweyh and coworkers, the internal consistency measured by Cronbach's alpha was 0.92 for the PSQ-total, 0.81 for the PSQ-minor, and 0.91 for the PSQ-moderate [6].

*(2) Quantitative Sensory Testing*. After completing the self-administered PSQ, participants waited until a researcher was able to perform QST. The pain thresholds were assessed in the healthy volunteers in reaction to painful electrical and pressure stimuli based on the Nijmegen–Aalborg screening quantitative sensory testing as described in earlier published research [25–27]. The twelve operators were trained thoroughly in the application of both stimuli in a training session for this study. In the training session, application of both stimuli was optimized and calibrated, as well as a uniform approach of participants. Instructions were standardized and read to each volunteer from an instruction sheet. To minimize distraction, participants were seated at tables in one of six separated cubicles facing the operator who was carrying out the measurements to create a low diverting environment and create the same ambient conditions. Moreover, protective industrial earmuffs (3M Peltor Optime III H540, Maplewood, MN, USA) were placed over the ears in order to minimize the influence of background noise. There were two cubicles for electrical stimuli and four cubicles for pressure stimuli. Measurements were performed on predetermined sites on the dorsal side of the nondominant forearm [28, 29]. The rationale for this location was based on the choice of performing the measurements on an easy assessable location with enough muscle size to measure the PPT. Moreover, this is also a location which is approachable by only uncovering the forearm but without undressing of the volunteer before performing the measurements. Participants were randomized to either the electrical or the pressure stimulus by picking a blinded envelope containing the protocol. First, a test measurement was performed to acquaint the participant with the procedure (M0). This was followed by the experimental stimulus used for analysis (M1).

*(3) Electrical Pain Tolerance (EPT)*. The EPT is the level of pain caused by an electrical current (expressed in milliampere, mA) which is as high as the participant could tolerate. To measure the EPT, the QST-3 device (JNI Biomedical ApS, Klarup, Denmark) was used. For the electrical pain stimuli, the electrodes (Kendall ECG Electrodes, H34SG, 50 × 45 mm; Covidien, Mansfield, MA, USA) were fixed at 10 and 6 centimetres from the styloid process of the ulna on the dorsal side of the nondominant forearm. The stimulator was set to deliver tetanic stimulation (100 Hz, 0.2 ms square waves) with a ramping rate of 1 mA/s. The initial current was set to 0 mA. For safety reasons, the maximum electrical current was limited to 50 mA. The participant was instructed to press a button to start the current and to release the button when the pain became intolerable.

*(4) Electrical Pain Tolerance Numerical Rating Scale (EPT-NRS)*. Participants were asked to rate the pain intensity experienced during the electrical pain tolerance, using the numerical rating scale (NRS) directly after the test. The level of pain was expressed on a NRS from zero (no pain) to ten (worst imaginable pain). The psychometric properties of the NRS are well supported, and the NRS is considered to be responsive and able to detect sex differences in pain intensity [30].

*(5) Pressure Pain Threshold (PPT)*. To measure the PPT, a digital pressure algometer with a 1.0 cm^2^ probe (Wagner Instruments, Force TEN^TM^ Digital Force Gage FDX 50, Greenwich, CT, USA) was used. The measurement locations were at 12 and 10 centimetres from the styloid process of the ulna and corresponded with the two measurements (M0, M1). The pressure was delivered under a 90° angle on the muscles on the dorsal side of the nondominant forearm. We used a ramping rate of ∼5 N/s by manually adjusting the applied pressure via visual feedback on the algometer display. PPT was expressed in Newton (N). Pressure started at 0 N and was applied up to a maximum of 250 N for safety purposes. The participants were instructed to say “stop” when they felt, besides the feeling of pressure, a burning, painful, or stitching sensation.

*(6) Pressure Pain Threshold Pain Intensity Rating*. Participants were asked to rate the pain intensity experienced during the pressure pain threshold, using the NRS directly after the test.

*(7) Statistical Methods*. Age and the PSQ-scores as well as the experimental pain scores were summarized as mean (standard deviation (SD)) and/or median (interquartile range (IQR)). Relative frequencies were calculated for nominal variables, such as sex.

The PSQ-total, PSQ-minor, and PSQ-moderate scores were evaluated for reliability by internal consistency using Cronbach's alpha. Since twelve researchers performed electrical and pressure pain tests, we tested for operator effects, using one-way ANOVA, with Tukey's post hoc testing with Bonferroni correction for multiple comparisons. Construct (convergent) validity was tested by evaluating the correlations between the PSQ and the experimental pain measures using Spearman's rank correlation coefficient [31]. Partial correlation coefficients between PSQ-scores and experimental pain scores, adjusted for age and sex as potential confounders [14], were calculated.

Data were analyzed using SPSS (IBM Corp. Released 2013. IBM SPSS Statistics for Windows, Version 22.0, Armonk, NY: IBM Corp.) and *R* (R version 3.4.0; *R* Foundation for Statistical Computing, Vienna, Austria). For all tests, *p* value <0.05 was considered statistically significant.

## 3. Results

### 3.1. Participants and Baseline Characteristics

**Figure 2:**
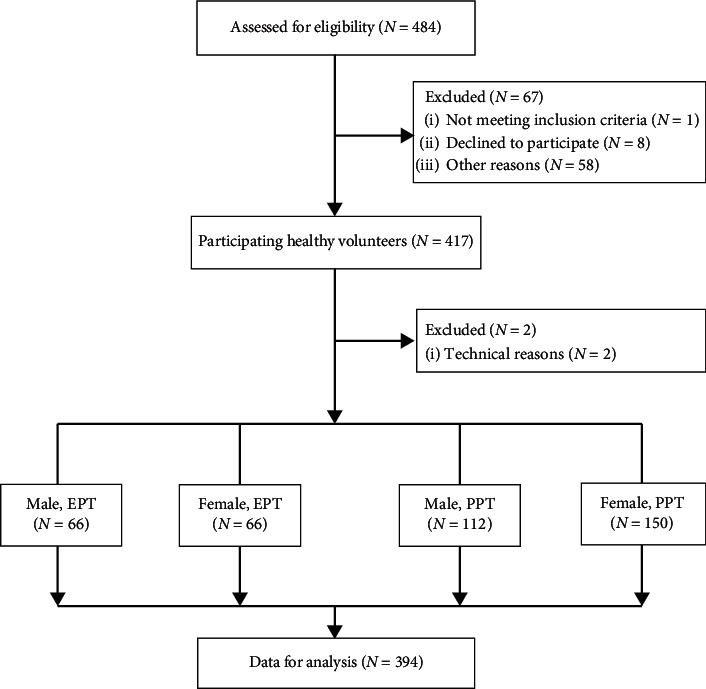
Flow diagram of the validation study. EPT: electrical pain tolerance measurement; PPT: pressure pain threshold measurement.

The enrolment process is shown in Figure 2. A total of 484 volunteers were assessed for participation. 67 volunteers were excluded, because they failed to meet the inclusion criteria (*n* = 1); declined to participate (*n* = 8); had drunk alcohol (*n* = 39); had taken recreational drugs (*n* = 10); had a painful arm (*n* = 1); had an open wound on their arm (*n* = 1); suffered from neurological (*n* = 1), psychiatric (*n* = 1), or cardiac diseases (*n* = 1); or had taken antidepressant (*n* = 2) or analgesic drugs (*n* = 2). Data from an additional 23 participants were excluded after performing the experiment. During data entry, the data of two participants were excluded from the study as it was unclear if they had the electrical or pressure pain test. During analysis, the data of 21 participants were excluded because of missing data on sex or the PSQ. The data of the remaining 394 subjects were used for the final analysis. 132 and 262 participants took part in the electrical and the pressure pain test, respectively, which was due to the availability of the measurement equipment.

A small majority of the total cohort were female (54.8%), and the median age of the participants was 26 years (IQR 22–32; range 18–60 years). The distribution of age and sex of the study participants is shown in Table 2.

### 3.2. PSQ-Scores

Scores of the Dutch PSQ are reported in Table 2. For PSQ-total, -moderate, and -minor, the mean was, respectively, 4.1, 5.3, and 2.8. Reliability was measured by internal consistency. Cronbach's alpha was 0.90 for PSQ-total, 0.86 for PSQ-moderate, and 0.82 for PSQ-minor.

### 3.3. Experimental Pain Testing

Table 2 shows the results of both electrical and pressure pain testing. Regarding the analysis of operator effects, there were no statistically significant differences between group means of EPT as determined by one-way ANOVA (*p*=0.496). Regarding PPT, the analysis revealed a statistically significant difference (*p* < 0.001) in the group mean of one operator compared to some other operators caused by some extreme values in PPT data. Therefore, we performed a sensitivity analysis by repeating all analyses without the data of this operator. No meaningful differences in the results were shown, so the results of the original data analyses are presented.

### 3.4. Construct Validity

**Figure 3:**
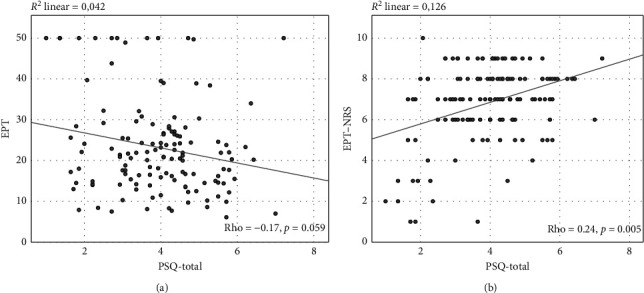
Illustration of the correlations between total score of the Pain Sensitivity Questionnaire (PSQ) and results of experimental electrical pain testing. (a) Correlation between the total score of PSQ (PSQ-total) and the electrical pain tolerance (EPT). (b) Correlation between the total score of PSQ (PSQ-total) and the electrical pain tolerance numerical rating scale (EPT-NRS). Linear regression lines are displayed, and Spearman's correlation coefficients (rho) as well as *p* values are given (*n* = 132).

**Figure 4:**
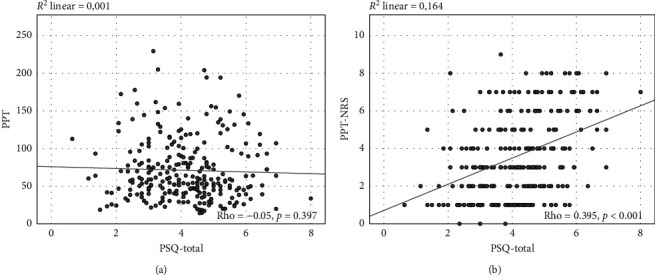
Illustration of the correlations between total score of the Pain Sensitivity Questionnaire (PSQ) and results of experimental pressure pain testing. (a) Correlation between the total score of PSQ (PSQ-total) and the pressure pain threshold (PTT). (b) Correlation between the total score of PSQ (PSQ-total) and the pressure pain threshold numerical rating scale (PTT-NRS). Linear regression lines are displayed, and Spearman's correlation coefficients (rho) as well as *p* values are given (*n* = 262).

Construct validity was evaluated by examining the correlations between the PSQ-Dutch and the experimental pain measures using Spearman's rank correlation coefficient. As shown in Table 3, no correlation was found between pressure pain threshold and any of the PSQ-scores. However, there was a statistically significant, but weak correlation between electrical pain tolerance and PSQ-moderate (rho = −0.19), and statistically significant weak to moderate correlations (rho = 0.22 to 0.40) between the PSQ-scores and the pain intensity ratings of both electrical and pressure pain tests (see Table 3 and Figures 3 and 4).

### 3.5. Confounding Variables

The experience of pain may be influenced by confounding factors; therefore, partial correlation coefficients between PSQ-scores and experimental pain scores, adjusted for age and sex, were calculated (see Table 3). As shown in Table 3, PSQ-moderate and PSQ-total, adjusted for age and sex, were weakly inversely correlated to EPT and showed statistical significance (PSQ-moderate: rho = −0.24; PSQ-total: rho = −0.22). No statistically significant correlation between PSQ-scores and PPT was found. Concerning the pain scores, all PSQ-scores were statistically significant and weakly to moderately correlated to EPT-NRS (PSQ-minor: rho = 0.21; PSQ-moderate: rho = 0.22; PSQ-total: rho = 0.23) as well as PPT-NRS (PSQ-minor: rho = 0.32; PSQ-moderate: rho = 0.36; PSQ-total: rho = 0.37).

## 4. Discussion

In this study, the PSQ was translated and cross-culturally adapted from both the English and German versions into the Dutch language. Furthermore, the instrument was validated in healthy volunteers. We found that the reliability via internal consistency of the PSQ-Dutch was high and that statistically significant correlations of weak to moderate magnitude were present between (1) the PSQ-moderate and PSQ-total and electrical pain tolerance and (2) all PSQ-scores and pain intensity ratings of both electrical and pressure pain stimuli. In contrast, there was no statistically significant correlation between PSQ and pressure pain thresholds. These relationships were maintained after controlling for age and sex.

We used the electrical pain tolerance as well as the pressure pain threshold [25, 26, 32]. Pain should be seen as a multidimensional, individual, and personal perception. Following our colleagues [33], it is therefore important that the response to a single, standardized stimulus is seen as a very limited part of the pain experience of a person. Because of this fact, we chose to use different stimuli (electrical and mechanical stimuli) and assessment techniques (threshold and suprathreshold response) to obtain more comprehensive information on nociceptive processing. As stated by Neziri et al. [34], both tests could be used for the assessment of pain hypersensitivity in patients.

The weak to moderate correlations we found in our study are comparable to those found in the original validation study as well as in the other language versions. Moreover, the reliability of the PPT and EPT was, based on other studies, acceptably high [35, 36]. However, the choice for electrical pain measurements in our study differs from the heat, cold, and pinprick measurements as used in the original study of Ruscheweyh [6] and in their validation study in patients with chronic pain [7], but the pressure pain algometry as an outcome measure was comparable between the studies of Ruscheweyh and our study. In the Mandarin Chinese version, similar to our study, electrical pain threshold and electrical pain tolerance were used [13]. In the Norwegian version, a comparison was made with the outcome of a heat pain threshold test and a cold pressor test [14]. In the English validation study, injection with lidocaine was used as a noxious stimulus [8]. The Polish [10], Korean [11], and the Iranian [12] studies regarding the validity of the PSQ did not use experimental pain sensitivity as a comparison, but other questionnaires, such as the Pain Catastrophizing Scale [37]. We believe that validation of a questionnaire reflecting an objective pain measure may be more reliable if tested with objective experimental pain measurement.

In our study, the score means of the PSQ were 4.1 ± 1.3 (PSQ-total), 2.8 ± 1.3 (PSQ-minor), and 5.3 ± 1.5 (PSQ-moderate) (see Table 2). The score means in the German version of the PSQ were somewhat lower: 3.6 ± 1.2 (PSQ-total), 2.5 ± 1.1 (PSQ-minor), and 4.7 ± 1.6 (PSQ-moderate) [6]. The score means of the Chinese version of the PSQ were a bit higher compared to the score means of the German and Dutch version of the PSQ: 4.7 ± 1.6 (PSQ-total), 3.9 ± 1.6 (PSQ-minor), and 5.5 ± 1.9 (PSQ-moderate) [13]. The differences between the PSQ-scores in the countries as stated above might be due to cultural differences or differences in the populations of healthy volunteers. Moreover, larger group inhomogeneity and differences in people who were carrying out the measurements might have played a role.

Construct validity was demonstrated by the presence of correlations between the results of experimental pain tests and the PSQ. It has been previously reported that the PSQ shows little or no correlation with experimental pain thresholds but substantial correlations with experimental pain intensity ratings, and this has been interpreted within a framework of pain thresholds and pain intensity ratings representing two different dimensions of pain sensitivity [6, 7]. Consistently, the present results show no statistical significant correlation between PSQ and PPTs, but statistical significant correlations between the PSQ and pain intensity ratings of both experimental pain stimuli used.

The relation between experimental pain tolerance and the PSQ has been less explored. One previous study reported correlations around *r* = −0.2 between PSQ-scores and electrical pain tolerance, which is very similar to our results [13]. There are however some differences between the measurements by Quan et al. [13] and our methodology. We used an alternating current (AC), whereas Quan et al. used a direct current (DC), and this difference is responsible for the much higher level of current in our study because the sensation of AC and DC is different. Moreover, our participants were able to abort the current by themselves instead of by the operator, which might give the participant more feeling of control. In addition, we included a bigger and probably more homogeneous population of participants. This all might have led to the differences in the current intensity between both studies but also makes both studies less comparable.

The perceived pain intensity during the measurement of the electrical pain tolerance was in the study of Quan et al. (via VAS) 6.21 ± 2.02. We found for the same assessment modality a pain intensity of 7 (IQR 6–8), which seems comparable. The present results show that the PSQ-Dutch represents mainly the pain intensity rating dimension of pain sensitivity, as previously shown for the original German version [6].

The strengths of this study are firstly the cross-cultural adaptation performed via an internationally established guideline. Secondly, the pain sensitivity measurements were adequately standardized and trained in beforehand. Moreover, the study population consisted of a large sample of both men and women.

In this study, we noticed some weaknesses. In our study population, we noticed that most study participants had an age between 18 and 38 years, thus leading to a rather homogeneous study sample of healthy volunteers. We also found that the median PPT of one of our operators was statistically significantly different, due to some extreme values of PPT data. These extreme values however were valid and reliable data, and the removal of the data of this operator made no substantive difference in the results. Therefore, we chose not to remove them from our dataset to avoid the risk of selection bias and type 1 error [38, 39]. Because of the study set-up during a three-day event, test-retest reliability could not be assessed. An interval of preferably one to four weeks, which is considered to be reflective for daily life and/or of clinical relevance, could not be reached. To minimize the burden due to pain stimuli and length of the total procedure for the participants, only one kind of pain stimuli (PPT or EPT) was given. In the validation studies of the English [8] as well as the Chinese version of the PSQ [13], also only one outcome modality was assessed. However, measuring only one modality might lead to a limited view of differences in pain sensitivity between patients as correlations of the PSQ with other types of experimental pain testing (cold pressor test, heat, pin prick stimuli, etc.) might be different. Furthermore, our exclusion criteria included pain of the arm, neck, or shoulder. Additionally, systemic diseases, which might have their influence on pain sensitivity (e.g., neurological, cardiac, and/or psychiatric diseases), were also excluded beforehand. We did not ask for other pain in the body. As any pain could have impact on pain sensitivity and thus influence the measurement outcome, this might have influenced the results.

## 5. Conclusion

Our study shows that the Dutch version of the PSQ was culturally appropriate for assessing self-reported pain sensitivity in Dutch adults. The instrument presented high internal consistency. Construct validity was similar to the original German version, with weak to moderate correlations between PSQ and experimental pain measures in healthy adult volunteers.

## Figures and Tables

**Table 1:** Comparison of items in the original German PSQ with the published English PSQ for cross-cultural translation into the Dutch language.

Item number	Original German PSQ	Published English PSQ	Final Dutch PSQ	Reasons for change
[Instructions]	Sie sollen dann entscheiden, ob diese situation für sie schmerzhaft wäre, und wenn ja, wie schmerzhaft sie wäre	You should then decide if these situations would be painful for you, and if yes, how painful they would be	Het is de bedoeling dat *u* van elke situatie bepaalt of deze pijnlijk voor *u* zou zijn en zo ja, hoe pijnlijk	*“Sie sollen”/*“*You should”* can be translated into the Dutch “*moeten,”* which is very demanding. Choice conforms to German PSQ formal language
[1–17]	Stellen sie sich vor, sie…	Imagine…	Stelt *u* zich voor dat	Conforming to the German PSQ usage of formal language
[10]	Stellen sie sich vor, sie haben eine kleine Verletzung am Finger und bringen aus Versehen Zitronensaft in die Wunde	Imagine you have a minor cut on your finger and inadvertently get lemon juice in the wound	Stelt *u* zich voor dat *u* een sneetje in uw vinger heeft en dat er per ongeluk citroensap in het wondje komt. Hoe pijnlijk zou dat voor *u* zijn?	Usage of diminutive for wound, which may feel as a very big wound in Dutch

**Table 2:** Characteristics of the participants.

	Total			Male		Female	
	*n*			*n*		*n*	
	394			178	(45.2%)	216	(54.8%)
Age (year)							
18–28				90	50.6	137	63.4
29–38				48	27.0	55	25.5
39–48				22	12.4	10	4.6
49–58				8	4.5	5	2.3
59–68				1	0.6	1	0.5
Missing				9	5.1	8	3.7

PSQ-minor (mean ± SD)		2.8	(±1.3)				
PSQ-moderate (mean ± SD)		5.3	(±1.5)				
PSQ-total (mean ± SD)		4.1	(±1.3)				

EPT (mA, median, IQR)	132	21.1	(15.0 – 27.1)				
NRS-EPT (median, IQR)		7.0	(6.0 – 8.0)				

PPT (N, median, IQR)	262	59.4	(41.6 – 92.2)				
NRS-PPT (median, IQR)		3.0	(2.0 – 5.0)				

SD: standard deviation; mA: milliampere; *n*: number of participants; EPT: electrical pain tolerance; PPT: pressure pain threshold; SD: standard deviation; NRS: numerical rating scale; IQR: interquartile range; N: Newton.

**Table 3:** Correlation between PSQ measures and QST measures (*n* = 394: EPT *n* = 132; PTT *n* = 266).

		EPT		EPT-NRS		PPT		PPT-NRS	
		Rho	*p*	Rho	*p*	Rho	*p*	Rho	*p*
PSQ-minor	Crude	−0.120	0.171	0.217	0.012	−0.074	0.235	0.349	<0.001
Adjusted for age and sex		−0.170	0.059	0.207	0.021	−0.106	0.097	0.324	<0.001
PSQ-moderate	Crude	−0.187	0.032	0.232	0.007	−0.032	0.611	0.378	<0.001
Adjusted for age and sex		−0.239	0.007	0.216	0.016	−0.044	0.490	0.363	<0.001
PSQ-total	Crude	−0.165	0.059	0.243	0.005	−0.052	0.397	0.395	<0.001
Adjusted for age and sex		−0.216	0.016	0.233	0.009	−0.078	0.222	0.374	<0.001

EPT: electrical pain tolerance; NRS: numerical rating scale; PPT: pressure pain threshold; *rho*: Spearman's rho; *p*: *p* value; PSQ: Pain Sensitivity Questionnaire.

## Data Availability

All data are available upon request to the corresponding author.

## Supplementary Materials

Supplementary Materials Dutch language version of the Pain Sensitivity Questionnaire (PSQ-Dutch).
