# Supplementary material for: Translation, Cross-Cultural Adaptation, and Validation of the Pain Sensitivity Questionnaire in Dutch Healthy Volunteers
Source: Pain Res Manag. 2020 Jul 23;2020:1050935. doi: 10.1155/2020/1050935 (PMC7397447; doi:10.1155/2020/1050935)
Supplement: Supplementary Materials — Dutch language version of the Pain Sensitivity Questionnaire (PSQ-Dutch). [file 1050935.f1.docx]

**Supplement S1: Dutch language version of the Pain Sensitivity Questionnaire** (PSQ-Dutch)

**Vragenlijst pijngevoeligheid**

Deze vragenlijst bestaat uit een aantal vragen waarbij u zich moet voorstellen dat u zich in een bepaalde situatie bevindt. Het is de bedoeling dat u van elke situatie bepaalt of deze pijnlijk voor u zou zijn en zo ja, hoe pijnlijk.

**Waarbij een 0 geen pijn betekent; een 1 nauwelijks waarneembare pijn en een 10 de ergste pijn die u zich voor kunt stellen of voor mogelijk houdt.**

Zet dan op de schaalverdeling een kruisje bij het cijfer dat voor u het meest van toepassing is. Houdt hierbij steeds in gedachten dat er geen ‘‘goede” of ‘‘foute” antwoorden zijn, het gaat slechts om uw persoonlijke beoordeling van de situatie.

Probeer zo min mogelijk uw angst of afkeer van de voorgestelde situaties van invloed te laten zijn op uw inschatting van de pijnlijkheid.

|  | | |
| --- | --- | --- |
| **1** | Stelt u zich voor dat u uw scheenbeen hard tegen een harde rand stoot, bijvoorbeeld tegen de rand van een glazen salontafel. Hoe pijnlijk zou dat voor u zijn?  0 = Helemaal niet pijnlijk 10 = de ergste pijn die u zich kunt voorstellen  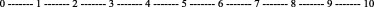 |  |
| **2** | Stelt u zich voor dat u uw tong brandt aan een gloeiend hete drank.  0 = Helemaal niet pijnlijk 10 = de ergste pijn die u zich kunt voorstellen  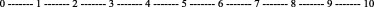 |  |
| **3** | Stelt u zich voor dat u lichte spierpijn heeft als gevolg van lichamelijke inspanning.  0 = Helemaal niet pijnlijk 10 = de ergste pijn die u zich kunt voorstellen  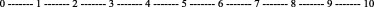 |  |
| **4** | Stelt u zich voor dat uw vinger klem komt te zitten in een lade.  0 = Helemaal niet pijnlijk 10 = de ergste pijn die u zich kunt voorstellen  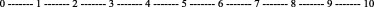 |  |
| **5** | Stelt u zich voor dat u douchet met lauw water.  0 = Helemaal niet pijnlijk 10 = de ergste pijn die u zich kunt voorstellen  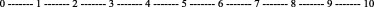 |  |
| **6** | Stelt u zich voor dat uw schouders licht verbrand zijn.  0 = Helemaal niet pijnlijk 10 = de ergste pijn die u zich kunt voorstellen  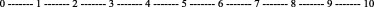 |  |
| **7** | Stelt u zich voor dat u van uw fiets bent gevallen en daarbij uw knie geschaafd heeft.  0 = Helemaal niet pijnlijk 10 = de ergste pijn die u zich kunt voorstellen  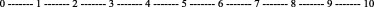 |  |
| **8** | Stelt u zich voor dat u tijdens het eten per ongeluk hard op uw tong of wang bijt.  0 = Helemaal niet pijnlijk 10 = de ergste pijn die u zich kunt voorstellen  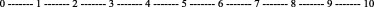 |  |
| **9** | Stelt u zich voor dat u met blote voeten over een koude tegelvloer loopt.  0 = Helemaal niet pijnlijk 10 = de ergste pijn die u zich kunt voorstellen  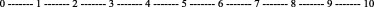 |  |
| **10** | Stelt u zich voor dat u een sneetje in uw vinger heeft en dat er per ongeluk citroensap in het wondje komt.  0 = Helemaal niet pijnlijk 10 = de ergste pijn die u zich kunt voorstellen  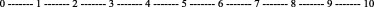 |  |
| **11** | Stelt u zich voor dat u uw vingertop prikt aan de doorn van een roos.  0 = Helemaal niet pijnlijk 10 = de ergste pijn die u zich kunt voorstellen  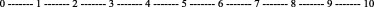 |  |
| **12** | Stelt u zich voor dat u uw blote handen een aantal minuten in de sneeuw houdt of dat uw handen een tijdje in contact met sneeuw zijn, zoals bij het maken van een sneeuwbal.  0 = Helemaal niet pijnlijk 10 = de ergste pijn die u zich kunt voorstellen  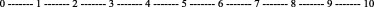 |  |
| **13** | Stelt u zich voor dat iemand u een normale handdruk geeft.  0 = Helemaal niet pijnlijk 10 = de ergste pijn die u zich kunt voorstellen  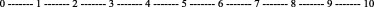 |  |
| **14** | Stelt u zich voor dat iemand u een zeer stevige handdruk geeft.  0 = Helemaal niet pijnlijk 10 = de ergste pijn die u zich kunt voorstellen  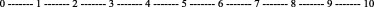 |  |
| **15** | Stelt u zich voor dat u per ongeluk een hete schaal oppakt bij de al even hete handvaten.  0 = Helemaal niet pijnlijk 10 = de ergste pijn die u zich kunt voorstellen  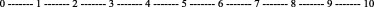 |  |
| **16** | Stelt u zich voor dat u sandalen draagt en dat iemand met zware laarzen op uw voet gaat staan.  0 = Helemaal niet pijnlijk 10 = de ergste pijn die u zich kunt voorstellen  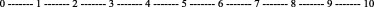 |  |
| **17** | Stelt u zich voor dat u uw elleboog tegen de rand van een tafel stoot (‘‘elektrisch botje”).  0 = Helemaal niet pijnlijk 10 = de ergste pijn die u zich kunt voorstellen  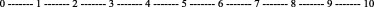 |  |
